# Cultural Humility Training in Mental Health Service Provision: A Scoping Review of the Foundational and Conceptual Literature

**DOI:** 10.3390/healthcare13111342

**Published:** 2025-06-04

**Authors:** Mayio Konidaris, Melissa Petrakis

**Affiliations:** 1 Social Work, Victoria University, Melbourne 3011, Australia; 2 Social Work, Monash University, Melbourne 3162, Australia; melissa.petrakis@monash.edu; 3 Mental Health Service, St Vincent’s Hospital, Melbourne 3162, Australia

**Keywords:** cultural competency, cultural humility, race, racism, mental health, mental health services and education

## Abstract

Background: Ongoing access and equity concerns for culturally diverse populations in mental health warrant a shift from cultural competence to cultural humility training. This review aimed to systematically assess the breadth of conceptual and training literature in peer-reviewed publications drawn from PsycINFO, CINAHL plus, Google Scholar and Scopus, from 2007–2018, utilizing *cultural humility* as the key search term and its relevance to service provision. Methods: This method utilized a five-stage scoping review framework. Results: Results were that a total of 246 publications were extracted. Following employing an abstract review method and removing duplicates, this resulted in a full-text review of 56 publications. The emerging themes included the following: culturally informed conceptual frameworks; culturally diverse training approaches; racial inequalities in mental health services; culturally informed national and international perspectives; race and international transcultural mental health. Conclusions: Conclusions were that including cultural humility principles in service provision and training enables greater self-awareness towards racial bias and negative cultural stereotypes at both practice and organizational levels, ultimately aimed at enhancing mental health service provision by mitigating the structural barriers encountered by service users.

## 1. Introduction

Enhancing mental health service provision with Cultural and Linguistically Diverse (CALD) populations is complex and dynamic, and the ongoing access and equity concerns are representative of broader inequities requiring attention. Practitioners’ building of knowledge around specific cultural contexts, and the related values and attitudes held by service users, is relevant to practice, particularly in terms of learning about individuals’ migration journeys and histories. These cultural histories shape and influence culturally diverse persons’ mental health experiences and presentations. Whilst developing such a depth of understanding is critical to service provision, it is equally important for service providers to be mindful around the potential risk of cultural assumptions and negative stereotypes misrepresenting CALD populations and potentially leading to poor clinical outcomes, according to Gainsbury [1], “Cultivating a sense of safety and working with ease and calm with all clients is likely to have benefits for retaining clients… Gainsbury [1] highlight the need for therapists to be comfortable in culturally uncomfortable settings”.

There is growing awareness regarding the limitations of cultural competence and cultural responsiveness training, given that it is historically less focused on promoting service provider reflexivity in challenging racial bias and cultural assumptions, and hence the significance of open cultural dialogues. This is evident in the Australian domain, in the context of transitioning the transcultural mental health curriculum from a prescriptive approach to one promoting cultural dialogues and conversation [2,3,4,5] an anthropological perspective on cultural competence, which further encourages *Open Forum*, promoting open dialogue as an opportunity for clinicians to capture “the complexity and indeterminate nature of culture…(to)…facilitate clinical encounters characterized by openness and a willingness to seek clarification when patients present with unusual or unfamiliar complaints”.

Australia’s cultural diversity rate comprising 28% of the population born overseas is the highest rate in 120 years, and is predicted to rapidly rise to 32% by 2050 [2]. Moreover, the most recent Australian census data report higher rates of a total of 49%, which include those who have migrated (first generation) along with subsequent generations, whereby one or both parents are born overseas [3]. In spite of service reform initiatives, there is an ongoing underutilization of services for such consumers and the family members or carers [4]. These include a significant omission of this cohort from Australian mental health research, primarily due to low English proficiency [2].

This remains a common thread throughout the Australian and international mental health literature, emphasizing evident disparities for particular ethnic groups experiencing higher rates of hospitalization and longer lengths of stay in hospital [5]. Greater coercion experienced in the process of being hospitalized is evident, such as in the UK and U.S. with cohorts of descent [6,7]. A study on second-generation migrants living with mental illness mentioned implications for adopting a trauma-informed lens with culturally diverse communities in mental health practice, particularly where issues of race are featured [8]. A scoping review is well-positioned in contributing towards the knowledge required in enhancing mental health service provision to better address the disparities and inequities impacting on culturally diverse populations on both individual and organizational levels. The breadth of scoping reviews is inclusive of a range of research designs and comprehensive syntheses, aimed at influencing policy and practice, other relevant programs and future study directions [9,10].

### 1.1. The Role of Social Work

Social work is well positioned to influence cultural contextual issues, given its foundational philosophy related to the important role of advocacy and engaging in a process of critical self-reflection. The significance of addressing implicit racial bias, which is often present in the sub-conscience, is also aligned with the critical social work practice lens. Therefore the implications of enhancing self-awareness in mental health service provision include the following: enhanced and more accurate psychiatric assessments and diagnoses, leading to the implementation of more appropriate treatments; enhanced therapeutic engagement, trust and rapport-building may result in improved medication compliance; alleviating the propensity of triggering additional depression and anxiety symptoms commonly associated with implicit racial bias in organizations also known as setting specific racism [11]; enhanced satisfaction with service provision for the provider and recipient; and attending to cultural influences in recovery at the exclusion of forming negative cultural assumptions and stereotypes [7]. Building such relationships of trust, rapport, and emotional safety with culturally diverse populations in service provision provides opportunity for greater therapeutic validation and, where relevant, increases the likelihood of addressing past or present experiences of racism.

### 1.2. Adopting a Cultural Humility Lens

As already highlighted, the founding of social work in critical self-reflexivity is well aligned with the construct of cultural humility. This is not a process attained by merely focusing on the external, but instead one that is internal and reflective, enhancing cross-racial dialogue and racial awareness. Adopting the notion of cultural humility endorses self-reflection as a critical tool towards enhancing racial literacy and challenging the barriers that act to maintain a divide between service providers and those from culturally diverse backgrounds. According to Qureshi and Collazos [12], the practitioner’s obligation and efforts to be non-racist and uncomfortable around racial themes further contribute to the manifestation of such issues within the therapeutic relationship. There is a growing body of Australian literature focusing on enhancing culturally competent care and addressing access and equity issues in the health of Aboriginal and Torres Strait Islander people. There is already relevant expertise, knowledge and learning available in relation to addressing such health inequities in clinical practice, training and other initiatives, including enhancing de-colonizing intercultural dialogue practices by Aboriginal (and non-Aboriginal) Australian academics and health professionals [13,14,15,16].

In Australia, few studies have explored the variables contributing to the complex reciprocal and relational dynamics between those from minority and dominant cultures, positioned within the mental health service context and influencing bias and stereotypes, and hindering better treatment outcomes. This review aims to inform a better understanding of the persisting complexities in inequities encountered by CALD populations.

## 2. Method

### 2.1. Search Strategy

To provide a solid historical foundation in the literature, a broad and extensive search was conducted focusing on peer-reviewed publications from January 2007 to January 2018. Employing the scoping review methodology enabled a focus on identifying broader pedagogies in health inclusive of cultural humility frameworks and learning from interdisciplinary literature [5,17], and thus, was not necessarily aimed at effectiveness. This is reflected in the research question, utilizing Arksey and O’Malley [9] five-stage scoping review framework, which includes identifying the research question, identifying relevant studies, study selection, charting the data, and collating, summarizing and reporting the reviews’ results. Firstly, the following is the research question of this review:

What is known about cultural humility and related concepts in the literature regarding mental health service provision and training?

Secondly, in identifying relevant studies, research evidence was drawn from the common social and psychological sciences databases of PsycINFO, CINAHL plus, Google Scholar and Scopus databases, given their association with psychological, medical, social and behavioral sciences, as well as nursing and allied health.

### 2.2. Search Terms

**Figure 1:**
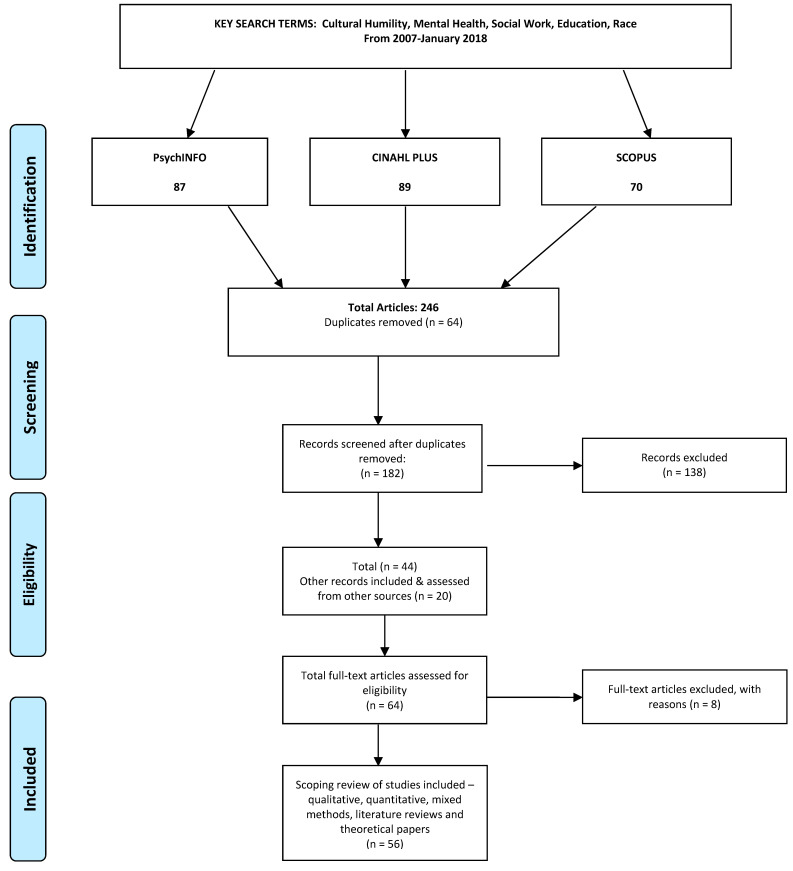
PRISMA 2009 Flow Diagram. From: Moher D, Liberati A, Tetzlaff J, Altman DG, The PRISMA Group (2009). Preferred Reporting Items for Systematic Reviews and Meta-Analyses: The PRISMA Statement. PLoS Med 6(7): e1000097. doi:10.1371/journal.pmed1000097 [18]. For more information, visit www.prisma-statement.org accessed on 16 March 2025.

A systematized method was adopted utilizing key search terms—cultural humility, mental health, race, social work, and education. Combinations of terms were utilized during this process, regarding mental health or mental illness, and the former concept prevailed as it enabled a broader mental health focus in the literature. Additionally, in order to enhance the transparency and completeness of the review, relevant sources in terms of other publications, reports, books and grey literature were also included, following reviews in the reference lists—refer to Figure 1 [18]. Furthermore, for the sake of data transparency and completeness, a PRISMA Checklist was also adhered to [19].

### 2.3. Inclusion and Exclusion Criteria

The following inclusion criteria have been used, aimed at exploring the relevant key theoretical constructs, training and pedagogy approaches specifically related to health and mental health settings, with a specific focus on issues related to race and racism: theoretical and review papers with specific reference to the concept of race and racism in health and mental health settings, central to education and training pedagogies; cross-disciplinary and inter-disciplinary research related to cultural humility frameworks; related concepts in health and mental health service systems; cultural humility principles applicable in diversity training and education related to mental health care; broader notions of cultural competence incorporating cultural humility; broader teachings drawn from ethno-specific research.

Furthermore, whilst elements of diversity overlap with cultural diversity, in order to privilege the broader theme of culture and cultural humility, and maintaining its centrality to this review, the exclusion criteria included the following: specific diverse populations (such as ethno-specific cultural groups and lesbian, gay, bisexual, transgender (LGBT) populations); broader notions of cultural competence (not inclusive of cultural humility principles) and cultural humility-related training within university settings and other general health, and non-health related settings; diversity training and education pedagogies not central to the cultural humility conceptual framework.

The fourth stage involves charting the data, which outlines key sources of information from the literature reviewed. The information gathered was charted utilizing an Excel spreadsheet in accordance with the following subheadings: authors, study focus, study populations, sample, methods, outcomes, and findings. Following this, the data were transferred onto a table, which offers a visual tool and a snapshot of the findings based on the described subheadings—refer to Table 1. Finally, the fifth stage below features the collation, summary and reporting of review results.

### 2.4. Data Items

A broad scope of data was extracted from qualitative, quantitative, mixed-methodological and theoretical studies. As this was a scoping review, the data obtained were predominantly descriptive via reading the full articles and identifying key methodologies and findings, reflecting the following characteristics: culture-related training in health and mental health services, including supervision models; lived experience of discrimination in service systems, with a particular focus on Aboriginal Australians and Indigenous communities in other international contexts; structural inequalities contributing to access for culturally diverse service users; culturally specific therapeutic interventions; and the role of relevant policies.

## 3. Results

The systematized search extracted a total of 246 publications within the period of January 2007–January 2018. While 64 duplicates were removed, 182 were screened utilizing an abstract reviewing method, and 138 publications were excluded, resulting in 44 articles. From other identified relevant sources, an additional 20 key articles from peer-reviewed publications were included. A full-text review of 64 articles resulted in an additional 8 being excluded. The scoping review screened a total of 56 publications, comprising qualitative, quantitative, mixed methodologies, general literature, and conceptual reviews, including theoretical papers—refer to Table 1. As a result, this method categorized emerging themes [69] following a thematic analysis, resulting in the following:

1. Conceptual frameworks around race, racism, institutional racism, cultural competence and cultural humility;
2. Culturally diverse training in health and mental health and the centrality of cultural humility;
3. Racial inequalities in mental health service provision—national and international perspectives;
4. Race, racism and the international transcultural mental health context.

### 3.1. Conceptual Frameworks—Race, Racism, Institutional Racism

Human bias and prejudice, and the propensity for racism and racial inequalities in health—deemed social health determinants—underlie the rationale for cultural humility training. Firstly, the concept of race is historically embedded in the social and anthropological sciences [28], and assumes biological and genetic differences in terms of visible and physical human characteristics. Race, however, “like gender is socially constructed…there really is no race under the skin and racism connotes the differences visible to people, which are a product of socialization, forming a racial lens and the significant ubiquitous impact on daily lives” [70]. Furthermore, DiAngelo [70] defines race as “The false concept that superficial adaptations to geography are genetic and biological determinants that result in significant differences among groups of human beings”.

As another socially constructed concept, whiteness studies are pertinent to the understanding of race and racism. Historically, culturally diverse groups in immigrant nations, such as the Irish, Italian and Polish, were not considered white, but due to processes of assimilation became white over time. Cultural groups that appeared white tended to be treated advantageously in society, thus assuming a position of power and privilege. According to DiAngelo [70], regarding immigrants of either the first or second generation holding a strong sense of ethnic identity compared to those of the same heritage who have been in the country for many generations, by virtue of looking white, they will still have a different external experience. This inconsistency between their internal senses of who they are, related to their ethnic identities and how they are racially observed on the outside as being white, results in a more “complex or nuanced sense of identity than someone who doesn’t question their whiteness. However, they will still be granted white privilege in dominant culture, and if they are committed to antiracist practice, they will need to explore how their white privilege shapes them” [70]. DiAngelo [70] defines whiteness as follows:

“A term to capture all of the dynamics that go into being defined and/or perceived as white in society. Whiteness grants material and psychological advantages (white privilege) that are often invisible and taken for granted by whites”.

Furthermore, the scientific understanding of racism has not been consistent over time [71]. DiAngelo [70] emphasizes racism significantly as both politically and emotionally fueled within society, stating that for whites, although they often have an opinion about racism, it is an issue that is commonly misunderstood. Inherently, racism reflects an individual’s perceived criticism, stemming from differences related to their physical or facial characteristics such as skin color, religion, attitudes, and value differences related to ethnicity and cultural heritage. Such experiences are particularly significant when members of the dominant culture perpetrate—either consciously or unconsciously, or even unknowingly (known as ‘unintentional racism’)—acts of assault, criticism and judgment towards a minority culture due to racial differences and culturally negative stereotypical perspectives. According to Helms [55], racial categories do not influence behaviors, though race appears in the therapeutic dialogue and impacts outcomes. Sue, et al. [25] indicates micro-aggressions manifest intentionally (or not), overtly or covertly in everyday life, and in particular in therapeutic practice defined. They can be identified as:

“…brief, everyday exchanges that send denigrating messages to people of color because they belong to a racial minority group. In the world of business, the term ‘microinequities’ is used to describe the pattern of being overlooked, under-respected, and devalued because of one’s race or gender. Microaggressions are often unconsciously delivered in the form of subtle snubs or dismissive looks, gestures, and tones.”

Owen, et al. [49], Hook, et al. [62], examined the association between microaggressions and the therapeutic working alliance, highlighting a correlation between the two. According to Owen, Tao, Imel, Wampold and Rodolfa [49], even though microaggressions are generally not discussed in therapy, they generally leads to weaker alliance or engagement if not discussed, compared to those who did not experience microaggressions, or who experienced them but discussed them. According to Hook 2016 [62], a counsellor showing cultural humility can result in fewer microaggressions, thus emphasizing the signficance of cultural humility principles in culturally diverse engagement and practice. Dudgeon, Wright, Paradies, Garvey and Walker [13] describe the individual’s perception or experience of racism as “self-reported” racism, whereas racism occurring between people is commonly defined in the literature as “interpersonal racism”. Strong emotions are often affiliated with the notion of racism depending on its context and how it is experienced or perceived. In modern multicultural societies with longstanding histories of migration, acts of racism towards minority groups are generally frowned upon, regardless of the sincerity of this condemnation or whether it is merely fueled by political correctness. However, racism is not always overt—it is also complex, insidious, and multifaceted. For racism that is not conscious, implicit association tests are commonly used to identify it, aiming “to measure the unconscious cognition of racist experiences” [40].

French writer and essayist of Tunisian–Jewish origin Memmi [72] has made efforts to discuss the complexities of racism, resulting in the following conclusions:

“Racism is a generalizing definition and valuation of differences, whether real or imaginary, to the advantage of the one defining and deploying them [accusateur], and to the detriment of the one subjected to that act of definition [victime], whose purpose is to justify (social or physical) hostility and assault [aggression].”

Miller’s paper [22] on the Web of Institutional Racism reports key covert and overt structural inequalities and barriers in the contexts of many fields. In relation to the mental health system, these are evident in policy and service provision, with theoretical bias influencing practice and non-inclusive culturally appropriate services. In the UK and Canadian transcultural mental fields, Fernando [27], Sashidharan [73], and colleagues have paid significant attention to issues of racism embedded in systems themselves, particularly with regard to the psychiatric service system. They argue that, despite services’ efforts over time to become increasingly culturally sensitive, issues of “institutional racism” within psychiatry remain unaddressed. The concept of “institutional racism”, as cited in Fernando [74] below, triggers questions requiring further exploration. He defines it as follows:

“The collective failure of an organization to provide an appropriate and professional service to people because of their colour, culture or ethnic origin. It can be seen or detected in processes, attitudes and behaviour, which amounts to discrimination through unwitting prejudice, ignorance, thoughtlessness and racist stereotyping which disadvantages minority ethnic people”.

According to Singh [24], however, the concept of institutional racism in mental health raises contentiousness and potential harm. This is further supported by the conclusions of Murray and Fearon [23] that racism in isolation is not the precursor to a high prevalence of psychosis, in particular among Africans and Caribbeans in the UK. This potentially “discounts the stressful impact and burden of busy caseloads and effects on staff, causing lowered employee satisfaction, internalizing blame of such systemic issues and feeling a sense of disempowerment to shift or change things…[emphasizing] that the concept of institutional racism is about shooting the messenger” [24]. Nevertheless, despite the contentious arguments around the validity of institutional racism in the mental health system, further attention to this issue is warranted in order to address the perpetual structural inequities in service systems for culturally diverse populations.

### 3.2. Conceptual Frameworks—Cultural Competence and Cultural Humility

According to Cross, et al. [75], cultural competence “...involves systems, agencies, and practitioners with the capacity to respond to the unique needs of populations whose cultures are different than that which might be called ‘dormant’ or ‘mainstream’ American”.

Cultural competence reflects the broad umbrella definition aimed at achieving cultural sensitivity on many levels (micro and macro), in clinical practice and organizationally. Given its “lack of agreeable mutual definitions” [44], the much-contested paradigm is explored in the context of cultural humility [1,47,57,75]. Cultural competence aims to provide a framework in order to address cultural diversity and equity concerns [38,44]. Cultural safety and cultural humility are considered significant in terms of building solid cultural competence foundations [38].

Kirmayer [41] reports challenges in promoting cultural competence in relation *to evidence-based practice* principles given that the framework itself has arisen from research samples not representative of culturally diverse cohorts, and other limitations. Cultural humility and cultural safety are highlighted as “an added critique of cultural competence” [41]. The former refers to clinicians’ access to insider cultural knowledge, being respectful and open to clients’ explanatory models; and the latter to “the historical and political aspects of health care…acknowledging structural violence…and safe clinical encounters” [41]. In the mental health arena, striving towards the absence of cultural bias and any form of racism aids “cultural safety”, a metaphor originating from the New Zealand Maori context and Indigenous populations, but also of some relevance to the CALD cohort. Kirmayer [42] describes that “cultural safety ‘moves beyond the concept of cultural sensitivity to analyzing power imbalances, institutional discrimination, colonization and colonial relationships as they apply to health care (National Aboriginal Health Organisation, 2008, p. 3)’”.

According to Dean [76], an enhanced understanding of both culture and race, and heightened awareness of its dynamic impact on mental health service provision, is a shared responsibility between culturally diverse communities and service providers. The service provider is thus in a position of power and advantage, with culturally diverse populations in a less powerful and disadvantaged position, stating the following:

“The paradoxical combination of these two ideas—being ‘informed’ and ‘not knowing’ simultaneously—captures the orientation to one’s ‘lack of competence’ that I am suggesting is needed in cross-cultural work…Learning about the ‘actual situation requires humility and respect for the time and work required to achieve understanding and develop a common set of goals, and purposes” [76].

The concept of cultural humility originating from within the medical field in the United States is gaining growing recognition globally [77]. This concept promotes greater self-reflection in therapeutic practice with regards to issues of race and power differences, and is considered integral to practice, defying the notion of culturally competent practice [42,44,45,46,47,48,49,50]. According to Tervalon and Murray-Garcia [77], cultural humility represents an eternal commitment towards the following:

“self-evaluation and self-critique, to redressing the power imbalances in the patient-physician dynamic, and to developing mutually beneficial and non-paternalistic clinical and advocacy partnerships with communities on behalf of individuals and defined populations”.

Yeager and Bauer-Wu [46] and Mosher, et al. [66] further add to the cultural humility paradigm. In particular, Yeager and Bauer-Wu [46] provide an additional dimension of Mindfulness to further enhance awareness of self and others, in relation to cultural humility in the research context. On the other hand, Mosher, Hook, Captari, Davis, DeBlaere and Owen [66] suggest that the notion of cultural humility supports positive therapeutic relationships and managing differences, and that it is a process without end. In addition, Beagan [52] and Hernández-Wolfe [31] reinforce fundamental principles of cultural humility in relation to being multi-dimensional, addressing power relations and how this enables critical reflexivity. Hernández-Wolfe [31] research with Latino populations suggests that striving for cultural equity and dialogue in mental health is also paramount, suggesting “…a co-existence instead of binaries and oppositions where a view must dominate the other”.

### 3.3. Culturally Diverse Training in Health and Mental Health and the Centrality of Cultural Humility

A number of studies (N = 17) have contributed to culturally diverse models and frameworks, adopting a range of methodologies and pedagogies, inclusive of health practitioners from a range of disciplines. The training modalities and paradigms relevant to this review include dialogical processes; quantitative [21,54,68]; qualitative [21,32,56]; mixed methodologies [37]; single case study illustrations, conceptual analyses [34,45]; critical and systematic literature reviews [35,45,48,58,78] and other relevant frameworks arising from case illustrations and clinical supervision models [48,50,63,64,65,78].

Bezrukova, Jehn and Spell [35] describe diversity training, “as a distinct set of programs aimed at facilitating positive intergroup interactions, reducing prejudice and discrimination, and enhancing the skills, knowledge, and motivation of people to interact with diverse others [63]”. Kowal, Franklin and Paradies [45] model of cultural diversity training critique, however, is influenced by critical race theory, underpinning theories of whiteness and anti-oppression and emphasizing reflexive antiracism as a key principle, addressing racialized identities by mitigating underlying prejudices. As outlined earlier, given the scoping nature of this review, the effectiveness of these training modalities was not the primary focus, but instead identifying the breadth of culturally diverse methods utilized in training was.

### 3.4. Racial Inequalities in Mental Health Service Provision: National and International Perspectives

Inequalities in service provision for culturally diverse populations represent a common thread throughout the Australian and international mental health literature. In the extant literature, disparities are emphasized across culturally diverse populations from particular ethnic groups experiencing higher rates of hospitalization and longer lengths of stay in hospital, as well as greater coercion in the process of being hospitalized [5]. The following common themes influencing such inequities include the following: issues of stigma [79]; language differences [79]; CALD populations experiencing fear in terms of service providers breaching confidentiality [39]; non-Western explanatory models of mental illness [80]; inherently covert elements such as general fear and mistrust, and underlying themes of negative racial stereotyping by service providers [81].

A qualitative Australian study addressing the limited mental health service use for Chinese migrants outlined a number of such barriers, including discrimination, in terms of “being belittled by the doctor for their poor English and treated rudely by reception staff” [26]. Cultural strengths are emphasized by responding to such practical issues when utilizing ethno-specific understandings. Influenced by Chinese immigrant experience, a 2012 U.S. study drew upon Chinese philosophies and cultural values by adopting a QIAN approach. That is, they cited humbleness in order to promote and foster cultural humility training in the broader health service domain [36].

According to Chang, Simon and Dong [36], the “QIAN curriculum could improve practice and enhance exploration, comprehension and appreciation of the cultural orientations between healthcare professionals and patients…QIAN model is highly adaptable to other cultural groups…Incorporating its framework…may enhance cross-cultural clinical encounters.”

The relevant literature generally addressed racist and discriminatory practices in the general health field and to some extent the mental health field. Paradies, Truong and Priest [50] systematic review examined the extent and measurement of healthcare provider racism worldwide from a range of health-related databases, resulting in a total of 37 studies identified almost exclusively from the U.S., including health professionals from medical, nursing and allied health disciplines. “Statistically significant evidence of racist beliefs, emotions or practices among healthcare providers in relation to minority groups was evident in 26 of these studies” [63]. Similarly, ref. [51] and colleagues systematized review on cultural competence training models with a focus on 19 published reviews further emphasized the importance of greater rigor in such studies with drawing specific attention to provider and patient outcomes. Overall, the former study suggests despite interest in racial disparities in healthcare, knowledge around “healthcare-provider racism” and how to best evaluate it remains limited. In addition, multi-method studies are considered the most optimal as opposed to a single methodological approach in capturing both the depth and breadth of such proposed concerns in healthcare practice [63].

### 3.5. Race, Racism and the International Mental Health Context

On an international level, the addressing of issues of race and racism in mental health service provision has diversified. This is related to socio-political differences and, in some countries, is due to consequences and histories of colonization. In Moodley and Ocampo [82] Critical Psychiatry and Mental Health, a comprehensive overview is provided of how issues of race and racial inequalities are addressed in countries, such as the U.S., Canada, UK, South Africa and New Zealand. Unlike the outlined Western countries, the absence of the Australian context is notable. The reasons for this remain speculative and unknown; however, it is consistent with the identified gaps in addressing issues of race in Australian mental health service provision and training.

An Australian grounded theory study exploring experiences of second-generation migrants living with mental illness concluded that if experiences of migration and its effects are not explored in service provision, histories of trauma are likely to be missed [8]. Thus, given the correlation between racism and mental health (whether perceived or self-reported racism) amongst immigrant populations living with a diagnosed mental illness, understanding this may assist in unfolding histories of trauma. However, Goodstein [83] cautions against racial paradigm dominance in clinical practice, emphasizing “making racial self-awareness and racial microaggressions primary over other types of awareness we risk missing the integrity of clients’ stories…[and]…our dialogue about race and racism often blurs the distinction between racial group and culture and may not exactly convey how people identify with racial or cultural groups”. While there is merit in both arguments, there is consistency in the former theme in this literature; this is particularly relevant in relation to the significance of practitioners’ self-awareness around their own cultural biases or racist attitudes as key to culturally sensitive practice.

Flaherty and Meagher [84] related research argued that the design of psychiatric inpatient units meant they were well-positioned to explore the interface between cultural diversity, difference, and mental health service responses. Given this, the “fishbowl-like exposure and the required interracial communal living, is a place where the issue of racial bias cannot be ignored. The task of living and working together in harmony is an important one for psychiatric patients and staff” [84]. This research indirectly explored racial bias within psychiatric hospital facilities, with a focus on the African American population, and found African American patients discharged themselves against medical advice more than the White patients, and tended to receive less allied health outpatient support in comparison to Whites. African American treating team members indicated patients who shared their cultural background were discussed at longer time intervals, in comparison to their White counterparts. The reverse also occurred for White service providers and White patients, suggesting a significant difference between the two groups [84].

Where racism is underlying or at the fore, CALD populations’ prior experiences of racism may continue to have adversarial effects on engagement and therapeutic outcomes. In addition, the associated consequences may result in CALD service users approaching the mental health system with some level of trepidation, perpetuating delays in early intervention treatment and the delivery of quality therapeutic outcomes, and raising barriers of mistrust. Such elements of distress experienced by CALD service users may be further perpetuated if they are also exposed to ongoing discrimination and negative (as perceived by them) attitudes for merely being culturally and racially different. Given this, Dean [76] argues the importance of being “aware of our own cultural baggage and separate[ing] ourselves from it in so far as is possible so that it will not interfere with our efforts to get to know another” perpetuating bias and inequities.”

Addressing negative judgments of CALD service users for reasons of sheer “cultural difference” and limited or poor English language skills is paramount. This is related to the length of time they have lived in the host country while continuing to require the services of an interpreter. Further, CALD service users should not fear judgment or a negative attitude on the part of mental health staff if they are closely affiliated with a particular religion or faith due to the risk of being misunderstood. Such propensity towards cultural misunderstandings, value judgments and racist or bias attitudes does not merely pertain to the broader public. The presence and influence of racism and racist attitudes can be seen anywhere, including organizational settings, health, and mental health systems.

## 4. Discussion

The findings of this scoping review are based upon international studies within western societies shaped and influenced by higher migration rates. Given that the results highlight the limits of such research in Australia, this discussion will draw upon the parallels and key learnings that can be derived from the international findings in terms of their relevance to the Australian context, and more specifically, social work practice.

Importantly, although cultural experiences uniquely differ between Indigenous Australians, migrant and refugee communities, the relevance of such research has been influential in the field of mental health in Australia. Azzopardi and McNeill [59] outlined a number of tenets relating particularly to the significance of critical self-awareness and challenges to inherent colonization ideologies, and emphasized a strengths-based orientation, as is particularly relevant to culturally diverse populations dealing with additional layers of barriers and obstacles. Maintaining critical self-awareness relies on an ongoing commitment to paying attention to cultural biases and assumptions, and upholding a strengths-based approach in practice also assists to challenging these barriers. Practitioners are well positioned to uphold a lens whereby one’s culture is considered an asset as opposed to a deficit. This may also enable greater cultural attunement and a respect for one’s values and beliefs, which may be in opposition to those of the practitioner. Jackson and Samuels [33] introduced cultural attunement to social work practice with culturally diverse populations, indicating that “Attunement requires one’s ‘cultural humility’ and awareness and acknowledgement of individual and group based experiences of pain and oppression (Hoskins, 1999)” [33]. Finally, engaging in such reflective processes around cultural attunement and critical reflection is synonymous with social work and mental health recovery-oriented practices. Thus, these processes are reliant upon organizational policies and practitioners’ abilities to uphold client self-determination and person-centered principles.

Such notions, along with mindfulness, are critical to promoting greater self-awareness and openness in mental health practice with culturally diverse service users. Additionally, this is an important aspect of the social work curriculum and the continuing professional development of social workers via ongoing cultural training and clinical supervision. These processes have specific relevance to the social work profession, given social work’s strong association with the historical forced removal of Aboriginal Australians from their families. The 2020 Australian Association of Social Workers (AASW) Code specifically emphasizes the criticality of whiteness theory, and the role of colonization and white privilege in social work practice, addressing First Nation Peoples, Aboriginal Australians and the significance of culturally competent practice to the social work profession [85]. Significant representatives within the Australian social work literature suggest the importance of White social workers developing a racial identity for them to perceive themselves as *raced*, and that this is socially constructed. Finally, given the significance of decolonizing Australian social work practice and enhancing racial knowledge, principles of cultural humility are significant in contributing towards a commitment to lifelong learning and greater self-awareness, and this is similar for non-White immigrant social workers.

The focus on “racism” as a social health determinant is steadily growing, in the public eye, in the literature, in the research and in training. Stolk, et al. [86] undertook a Victorian mental health study with a culturally diverse urban sample, and outlined factors contributing to the service access rates for CALD service users, suggesting “… mental health staff may be lacking in cross-cultural clinical competence, possibly misdiagnosing unfamiliar manifestations of mental disorder and resorting to involuntary admission when lacking confidence in their assessment of an NESC client (Minas et al., 1994; Stolk, 2005)”. The limitation in related Australian research is two-fold, regarding both the unaddressed CALD service users’ lived experiences of racism, and the service providers’ response to this. According to the research of Marsella [87], the consequences of racism for CALD youth may result in emotional and mental wellbeing problems, such as depression and anxiety. The young person may also be in a state of acculturation, navigating transitioning from one culture to the other, in terms of their sense of belonging and ethnic identity development. This is not only a vulnerable time for the young person and their family, but also one that is exacerbated by stress, vulnerabilities, and any forms of encountered racism. However, despite the rising level of evidence in this area, less is known about the histories and stories of racism encountered by those already living with a diagnosed mental illness, and their respective carers and families, including if self-reported racism for CALD individuals has manifested into consequences of trauma.

Finally, the emergence of a workforce with lived experience in mental health settings, both locally within the Australian context and internationally, such as in the UK, the U.S. and Canada, raises hope in strengthening the voices around race and racism for culturally diverse service users. To achieve this, it is vital for practitioners to engage in cultural attunement and critically reflective processes, while also being aligned with social work and mental health recovery-oriented practices. Emerging global shifts towards privileging the voice of lived experience, with attention to cultural and racial experiences, rely on the support of organizational systems and policies, and practitioners’ upholding of client self-determination and person-centered principles [85]. The incorporation of person-centered approaches is aimed at promoting and empowering service users’ needs and choices, including enhancing trust- and rapport-building and an authentic collaborative partnership, in order to enhance shared decision-making and culturally informed assessment and intervention processes [88,89].

## 5. Limitations

Whilst this review aimed to address the breadth and descriptive aspects of the relevant literature, the limitations are reflected in the lack of focus on the sources’ depth and quality [9], including discrepancies in concept definitions across the spectrum of the literature [90]. Further, as this review primarily aimed at focusing and centralizing cultural diversity, other relevant and diverse groups formed the exclusion criteria and warrant attention in future related studies.

## 6. Conclusions

Issues of racism and its effects are universal and transcend cultures. Given this, the notion of cultural humility continues to resonate worldwide in a range of fields, particularly in relation to education and health service provision. Cultural humility promotes an optimistic, challenging, and non-threatening approach towards the peeling back of layers posing as barriers in service provision. However, given that the notion of cultural humility is not the most commonly adopted construct globally, several inconsistencies and gaps are highlighted. These relate to the language or terminology utilized to describe training approaches aimed at enhancing the quality of practice and therapeutic outcomes with culturally diverse populations in service provision, and some consideration towards incorporating service-users’ voices may assist to further enhance cultural humility practice. This may result in perpetuating ‘othering’, and the pursuit of cultural training primarily focused on attaining cultural knowledge and not enhancing self-awareness.

To enhance service quality, clinical outcomes, access, and equity concerns for culturally diverse communities across the globe, adopting and fostering cultural humility principles is crucial in mental health service provision. Practitioners carry an ethical responsibility related to not merely being bound by cultural knowledge, but also in terms of holding this lens alongside their own self-awareness. This enables practitioners to ensure greater accuracy in assessment processes (and ultimately treatment outcomes), or at the very least to utilize their own curiosity and critical self-awareness to ‘check in’ with the service regarding the accuracy or inaccuracies of these assumptions.

In the Australian societal context, experiences of racism are their own entity and unique to each cultural group, although they arguably remain a common thread between migrant, refugee and Aboriginal Australians. Bennett, et al. [29] and Eley, et al. [20] emphasize collaboration and a process of the ‘joint development of knowledge’ between Aboriginal and non-Aboriginal social workers, poignantly prioritizing “knowing who you are culturally…and the ability to respond with humility and genuineness…” [29].

Whilst it would be somewhat idealistic to aim towards a set of common universal terms that should be adopted by health services, policy-makers and academic settings, perhaps a more realistic approach would be to incorporate or adopt language essentially aimed at challenging underlying racial bias and cultural assumptions, and explore ways to maintain an ongoing process of self-reflexivity; that is, to consider concepts or narratives relevant to an individual country’s historical, cultural and migration contexts. Finally, it is recommended that future research examine the effectiveness of such consistent frameworks within training and teaching contexts, inclusive of service-recipient voices.

## Figures and Tables

**Table 1:** Findings Table—Cultural Humility Training in Mental Health Service Provision—An Overview of the Foundational and Conceptual Literature.

Year	Author	Study Focus	Study Populations	Sample	Methods	Outcome/Findings
**2007**	Carpenter-Song, et al. [3]	‘Open dialogue’ in mental health care	Culturally diverse clients and practitioners	Nil	Promoting Dialogue	Culture pertinent to Culturally Competent Practice in Mental Health Care.
**2007**	Eley, D., et al. [20]	Services to Indigenous mental health service users	Staff; Indigenous services users, families & carers	n = 164 (staff); n = 126 (Indigenous)	Quantitative study—Questionnaires	Indigenous peoples’ needs not met by existing mental health services.
**2007**	Griswold, K., et.al [21]	Medical trainees’ self-awareness and refugees	Medical Trainees	n = 27	Qualitative	“Hands on” approach to cultural diversity training enhances cultural sensitivity.
**2007**	Miller, J., et al. [22]	Identifies major forms of institutional racism in the U.S	Social Workers and other helping professionals	Nil	Theoretical	Recognition of institutional racism essential for the helping professions.
**2007**	Murray, Fearon et al. [23]	Challenging notion of ‘Institutional Racism’ in UK mental health context	Nil identified	Nil	Commentary paper	There is no more racism in psychiatric settings than in broader society, failing to consider strengths in services.
**2007**	Singh, S.P. [24]	Institutional Racism in Psychiatry-Challenging notion	Mental Health System	Nil	Commentary paper	Institutional racism and discrepancies in higher admission rates for some cultural groups.
**2007**	Sue, D.W., et al. [25]	Racial dialogue between a White Counselor and Person of Colour	Dialogue dyad Therapist and Client Dyad	Single Case Design	Qualitative	Interracial encounter prone to the manifestation of racial microaggressions.
**2008**	Blignault, I., et al. [26]	Barriers to Mental Health Service Utilisation Chinese Migrants	Chinese Born Mental Health Clients	n = 9 services users; n = 1 (caregiver); n = 11 (service providers); n = 13 (community members)	Mixed methodology—quantitative & qualitative	Prevention & Treatment Implications for Native Born Chinese & heterogenous assumptions.
**2008**	Fernando, S. [27]	Cultural sensitivity and Race in training	Mental Health Service systems	Nil	Theoretical	Cultural Sensitivity over ‘cultural competence’; focus on race over culture in training.
**2009**	Gravlee, C.C. [28]	Social inequalities & racial inequalities service provision	Nil identified	Nil	Literature Review	Model to enhance understanding of how racial inequalities are embodied.
**2011**	Bennett, B., et al. [29]	National research project Findings-Social Work with Aboriginal People	Aboriginal Elders & Non-Aboriginal Social Workers	n = 19	Qualitative	Strengths towards developing a practice framework with Aboriginal people/communities.
**2011**	Furlong, M., Wight, J. [30]	Working cross-culturally and reflective self-scrutiny	Development Curriculum Content	Human Academic curriculum	Theoretical and conceptual Critique	Cultural competence achieved via critical self-reflection & cultural knowledge learning.
**2011**	Hernández-Wolfe, P. [31]	Introduces Latin American decolonising paradigm	Latinos	Single case illustration	Theoretical and single case illustration	Paradigm provides cultural lens to mental health integrating cultural equity/humility.
**2011**	Hoke, M.M., et al. [32]	Teaching cultural competency to graduate psychiatric nursing trainees	Post Graduate Psychiatric Nurses	Nil	Qualitative	Integrated culturally competent training approach & cultural humility is optimum.
**2011**	Jackson, K.F., et al. [33]	Social work practice with multi-racial populations	Multiracial People	n = 34	Critique-generating themes from literature	Culturally attuned approach inclusive of multi-racially is optimum in social work.
**2011**	Qureshi, A., Collazos, F. [12]	Cultural therapeutic relationship	Mental Health Clinicians & clients	Nil	Traditional & Modern Cultural Systems Frameworks	Significance of Culture-Immigration-Psychosocial Development/Skills/Attitudes/Beliefs.
**2012**	Ascoli, M., et.al [7]	Cultural Consultation to improve client outcomes	Mental Health Clinicians	Qualitative—narrative analysis	Cultural Consultation Approach	Useful tool in analysing the scale of problems, but limited in offering solutions.
**2012**	Baarnhielm, S., et al. [34]	Cross-cultural mental health training-German and Swedish contexts	Mental Health Clinicians	n = 3 (training programs)	Critique and scoping method	Significance of cross-cultural training inclusive of a cultural humility framework.
**2012**	Bezrukova, K., et al. [35]	Critical review of a range of diversity training formats	University Campus & Workplaces	n = 178 (Articles)	Critical Review Method	Rare and positive forms of integrated diversity training were more favourable.
**2012**	Chang, E.-S., et al. [36]	Integrating cultural humility into health care professional training	Health professionals	Nil	Qualitative—QIAN Model	Importance of integrating cultural humility in health training based on the QIAN principles.
**2012**	Conrad, S.C., et al. [37]	Describes a model for interprofessional and transcultural learning	Community Health Workers/Professionals &Trainees	n = 58	Mixed Methods—Qualitative Narratives	Transcultural & Interprofessional healthcare immersion enables shared learning.
**2012**	Fung, et al. [38]	Methodology assessing cultural competence-mental health organisations	Mental Health Organisations	n = 133 (Focus Group); n = 26; n = 238	Mixed methods	8 Major Domains proposed to comprehensively evaluate cultural competence.
**2012**	Hall, C.A., et al. [39]	African American services users dealing with barriers in therapy	African Americans	n = 9	Phenomenological Study	Common barrier of stigma, qualities of resilience & change recommendations in therapists.
**2012**	Harris, R., et al. [40]	Self-reported Racial Discrimination	Adults aged 15+ in New Zealand	n = 12,474 (2006/07)	Population Based Survey—Quantitative	Racial discrimination potential to impact on range of health outcomes & risk factors.
**2012**	Kirmayer, L.J. [41]	Mental health services incorporate culturally competent models	Relevant literature	Nil	Review of the relevant literature	Deconstructing cultural Competence, while considering cultural safety, sensitivity, and humility.
**2012**	Kirmayer, L.J. [42]	Evidence Based Practice (EBP) and Cultural Competence (CC)	Relevant literature	Nil	Literature and Conceptual Review	Clinical bias in EBP & cultural groups’ ‘ways of knowing’ possible discredited in mental health.
**2013**	Hook, J.N., et al. [43]	Cultural humility as an important quality in a therapist	College Students	n = 117	Quantitative Pilot Study	Cultural Humility’s positive association with therapy improvement/therapeutic working alliance.
**2013**	Horevitz, E., et al. [44]	Black box’ of cultural competence	Relevant literature	Nil	Literature review	Effectiveness of cultural competence and its association with reducing disparities.
**2013**	Kowal, et al. [45]	Reflexive Anti-Racism in Diversity Training	Theoretical Frameworks	Nil	Conceptual Review	Reflexive Anti-Racism as an alternative framework in diversity training.
**2013**	Poon, W.C., et al. [5]	Chinese caregivers living with Schizophrenia in Australia	Chinese caregivers	n = 5 (Caregivers); n = 7 (Professionals)	Mixed Methodological Design	Support modalities relevant to mental health social work, also focus on clinicians’ self-scrutiny.
**2013**	Yeager, K.A., et al. [46]	Cultural Humility research context	Researchers & migrant populations	Nil	Theoretical and conceptual critique	Cultural Humility practices needs to be essential in the training of researchers.
**2014**	Isaacson, M. [47]	Nursing student cultural competence perceptions	Nursing students	n = 11	Mixed methodology—quantitative & qualitative	Cultural humility approach is emphasised, within various perceptions of cultural competence.
**2014**	Murray-Garcia, J.L., et al. [48]	Open dialogue about race/racism	Health Professionals& Educators, Clients	Trainees in health services	Literature Review	Social psychology’s association with implicit/explicitly bias/racism, reversal of disparities.
**2014**	Owen, J., et al. [49]	Microaggressions impact on the therapeutic relationship?	University Counseling Clients	University counselling sample	Racial Microaggressions in Counseling Scale	Microaggressions present in therapeutic milieu, which majority not discussed in therapy.
**2014**	Paradies, et al. [50]	Review focusing on interpersonal racism amongst health care providers and measurement approaches	Almost solely US based with healthcare providers	Physicians and health care providers	Systematic Review	Statistically significant evidence of racist beliefs, emotions, and practices among health care providers towards minority cultural groups. Limited knowledge remains around health care provider racism and how to measure it.
**2014**	Truong, et al. [51]	Systemised Review focusing on Cultural Competence Training Models	Cultural Competence Models in Health	n = 19 (published reviews)	Systematised Review	More rigorous designs needed, focusing on provider and patient outcomes.
**2015**	Beagan, B.L. [52]	Culture and diversity in OT field from 2007-2014	OT Literature	110 articles	Literature Review	Cultural Safety & Cultural Humility preferable in approaching diversity & power relations.
**2015**	Fisher-Borne, M., et al. [53]	Cultural Competence & Cultural Humility as the alternative framework	Cultural Competence & Cultural Humlity Frameworks	Nil	Two model comparison	Conceptual Model of Cultural Competency, service provision also at organisational level.
**2015**	Hankerson, S.H., et al. [6]	Racism/discrimination/cultural mistrust/misdiagnosis/clinician bias	Depressed African American Men	Nil	Literature Review	Clinical and Community Entry Points to engage African American men are identified.
**2015**	Hayes, J.A., et al. [54]	Therapists’ effectiveness with culturally diverse clients	Clients and Therapists	36 Therapists & 228 Clients	Quantitative study	Therapists’ effectiveness varies; implications for training and practice.
**2015**	Helms, J.E. [55]	Effectiveness of cultural adapted practice	African/Asian/Pacific Islander Americans	Nil	3 Meta Analyses and 3 studies analysis	Greater focus on culturally adapted approaches by researchers and practitioners.
**2015**	Kools, S., et al. [56]	Culturally appropriate diversity program	Global Health Fellows	n = 25	Phenomenological Study—Qualitative	Useful learning related to cultural humility, particularly in an interprofessional framework.
**2016**	Aggarwal, N.K., et al. [57]	Meanings and practices of cultural competence	Service users, clinicians, and administrators	n = 30	Exploratory Focus Group—Qualitative Methods	Value of qualitative research in hospitals, and forms of barriers to care illustrated.
**2016**	Alhejji, H., et al. [58]	Diversity Training Outcomes Analysis	Relevant literature	Nil	Systematic Review—3 Perspective Approach	Improved research/methodologies required incorporating cross-disciplinary perspectives.
**2016**	Azzopardi, C., et al. [59]	Framework for culturally responsive social work practice	Practitioners working with diversity	260 Independent Samples	Cultural Consciousness Model	Model addressing social work practice with diversity across a range of levels.
**2016**	Bezrukova, K., et al. [60]	Diversity Training on 4 Outcomes over time	Relevant Training Models	Nil	Meta Analysis-Diversity Training Outcomes	Primary impact for training over time to focus attitudinal shifts & range of learning modalities.
**2016**	Foronda, C., et al. [61]	Concept Analysis and defining of Cultural Humility	Conceptual Literature	Multidisciplinary concept reviewers	Rodgers and Knafl’s (2000) method of concept analysis	Multidisciplinary input on concept analysis enabled diverse perspectives.
**2016**	Hook, J.N., et al. [62]	Microaggressions in counseling with perceived cultural humility	Adult population from the U.S.	N = 2212	Quantitative study	High proportion racial microaggressions in counseling & cultural humility reduced this.
**2016**	Watkins, C.E, et al. [63]	‘Cultural Third’ & Cultural Humility in Psychotherapy Supervision	Psychotherapists/Supervisors/Clients	2 Case Scenarios between Psychotherapist and the client	Case Examples	Positive outcome utilising Cultural Humility Principles.
**2017**	Clabby, J.F. [64]	Enter as an Outsider’ Teaching Program	Health Professionals	General Overview of Samples	Qualitative	Participants improved Cultural Humility understanding and re position as an Outsider.
**2017**	Foldy, E.G., et al. [65]	Group Relations Theory enhancing Cultural Competence	Clinical and Broader Organisational focus	Nil	Group Relations Theory	Group Relations approach in creating greater openness around racial issues.
**2017**	Gainsbury, S.M. [1]	Cultural competence in mental health & addiction disorder treatments	Mental health Clients with addictions	Nil	Rapid Evidence Review Method	Benefits shown in cultural competence within addiction treatment programmes.
**2017**	Mosher, D.J., et al. [66]	Applying Cultural Humility in Therapy	Counsellors-Therapists	2 x Therapist/Client Dyads	4 Part Framework	Higher Cultural Humility associated with a more therapeutic engagement and outcome.
**2017**	Owen, J., et al. [67]	Client ratings of therapists’ cultural humility and cultural identity	University Counseling cohort	N = 247 Treated by 50 therapists	Quantitative study	Despite no significant difference noted, multicultural orientation model is useful.
**2018**	McLennan, V., et al. [14]	A non-Indigenous researcher’s narrative reflection	Indigenous Populations	Single Case Study	Narrative Reflection—Use of Self	Indigenous Standpoint Theory is optimum, with Western & Indigenous Research Paradigms.
**2018**	Sempértegui, A.G., et al. [68]	Diversity-oriented competence training in clinical assessment	Mental Health Clinicians	n = 40	Quantitative	Population-specific, diversity oriented competence training increases related knowledge.

## Data Availability

Data is contained within the article. The original contributions presented in this study are included in the article. Further inquiries can be directed to the corresponding author.
